# Molecular genetic aberrations in the pathogenesis of multiple myeloma

**DOI:** 10.2478/abm-2023-0056

**Published:** 2023-10-18

**Authors:** Ivyna Pau Ni Bong, Ezalia Esa

**Affiliations:** Hematology Unit, Cancer Research Center, Institute for Medical Research, National Institute of Health, Ministry of Health, Malaysia

**Keywords:** genomic aberrations, molecular pathogenesis, multiple myeloma, mutational landscape, oncogenic events

## Abstract

Multiple myeloma (MM) is the second most common form of blood cancer characterized by clonal expansion of malignant plasma cells within the bone marrow. MM is a complex, progressive, and highly heterogeneous malignancy, which occurs via a multistep transformation process involving primary and secondary oncogenic events. Recent advances in molecular techniques have further expanded our understanding of the mutational landscape, clonal composition, and dynamic evolution patterns of MM. The first part of this review describes the key oncogenic events involved in the initiation and progression of MM, together with their prognostic impact. The latter part highlights the most prominent findings concerning genomic aberrations promoted by gene expression profiling (GEP) and next-generation sequencing (NGS) in MM. This review provides a concise understanding of the molecular pathogenesis of the MM genome and the importance of adopting emerging molecular technology in future clinical management of MM.

Multiple myeloma (MM) is characterized by the accumulation of malignant plasma cells in the bone marrow and excessive production of a single monoclonal immunoglobulin. MM is the second leading cause of blood cancer worldwide, which accounts for approximately 10% of hematologic malignancies [1]. A recent increase in the incidence of MM has been described in Asian countries such as Japan, Korea, and Taiwan [2,3,4]. MM is more likely to occur in elderly men [4]. Generally, patients with early-stage MM are without symptoms (asymptomatic); however, symptoms develop following disease progression. The most typical clinical manifestations of MM include hypercalcemia, renal failure, anemia, and bone fractures (CRAB symptoms) [5]. Over the past several years, treatment strategies have expanded extensively for MM patients. The development of new drugs has led to significant improvements in prognosis and overall survival (OS) among patients. Key drugs for treatment are immunomodulatory imide drugs (IMiDs) (thalidomide, lenalidomide, and pomalidomide), proteasome inhibitors (bortezomib, carfilzomib, and ixazomib), monoclonal antibodies (daratumumab and elotuzumab), immune-based therapies (CAR-T cell and bispecific T-cell engager [BiTE]), and small-molecule inhibitors targeting FGFR3 and RAS/MAPK signaling pathways (erdafitinib, vemurafenib, and cobimetinib) [6, 7]. However, despite recent advances in therapeutic approaches in MM, it remains incurable. Although most patients generally respond to the standard first-line treatment, many of them would inevitably relapse or become refractory to the disease [6].

Conventional karyotyping and fluorescence in situ hybridization (FISH) are the gold standard techniques used for detecting cytogenetic abnormalities in MM. Conventional karyotypes are prepared from mitotic cells that have been arrested at the metaphase stage. About 30–50% of cytogenetic changes in MM can be detected using karyotyping; however, they are frequently acquired from advanced cases [8]. Conventional karyotyping tends to miss chromosomal aberration in early stages of the disease, due to its low mitotic index and number of malignant plasma cells. The low resolution of conventional karyotyping has also limited the identification of cryptic, subtle, or complex chromosomal changes. FISH, on the other hand, is more sensitive than classical cytogenetics. FISH uses interphase nuclei and thus does not require cell proliferation. It uses fluorescence-labeled probes to detect specific DNA sequences on the chromosome. Consequently, up to 80% of chromosomal aberration in MM can be identified by FISH [9]. The sensitivity of FISH testing can be enhanced with plasma cell enrichment of bone marrow specimens. Nevertheless, the main disadvantage of FISH is that it can only detect known chromosomal aberrations. Recently, with the progress in molecular techniques such as gene expression profiling (GEP) and next-generation sequencing (NGS), various driver gene mutations, oncogenic dependencies, structural variants (SVs), chromoplexy, and chromothripsis in relation to MM clonality and evolution have been discovered. Although GEP and NGS have paved a way toward a more effective management of MM patients, most of the developing countries in Asia, including Malaysia, are still relying on conventional methods (G-banding karyotype and FISH) to detect structural and numerical chromosomal aberrations in MM. The current challenge is whether new emerging molecular techniques should be adopted in the routine clinical practice of MM to improve the diagnosis and management of MM patients.

In this review, we describe the recurrent primary and secondary oncogenic events in MM and their clinical impacts on the prognosis of patients. We also summarize the most prominent findings concerning genomic changes (molecular signatures, new and rare somatic mutations) contributed by GEP and NGS studies of MM. We compiled the relevant articles for this review from electronic databases, including PubMed, Google Scholar, Scopus, and Web of Science. Keywords used for searches included MM, translocation, primary and secondary oncogenic events, chromosomal abnormalities, gene expression, NGS, mutation, clonal evolution, genomic landscape, and SVs. All relevant articles were carefully reviewed, and more articles were searched based on the citation in the articles.

## Molecular events in the pathogenesis of MM

**Figure 1.**
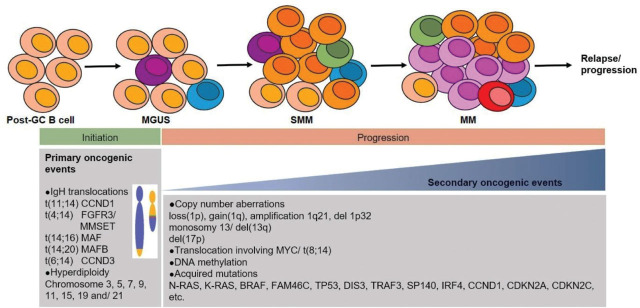
Primary and secondary oncogenic events in the molecular pathogenesis of MM. MGUS, monoclonal gammopathy of undetermined significance; MM, multiple myeloma; SMM, smoldering multiple myeloma.

MM is a heterogeneous and genetically complex disease. It is thought to occur via multiple primary and secondary oncogenic events (**Figure 1**) [10]. Hyperdiploidy and translocations involving the IgH gene rearrangement occurring during early B-cell development in germinal centers are primary oncogenic events that initiate the development of MM [11]. The most common primary chromosomal abnormalities in MM are hyperdiploidy, translocations t(4;14)(p16;q32), t(14;16)(q32;q23), t(14;20)(q32;q11), and t(11;14)(q13;q32). Secondary oncogenic events involve copy number aberrations (CNAs) such as monosomy or del(13q), del(1p), gain(1q21), del(17p)/*TP53*, *MYC*-associated translocations, and acquired mutations (*K-ras*, *N-ras*, *BRAF*, *TP53*, etc). Secondary oncogenic events aggravate disease progression and frequently develop at the later stage of the disease [11]. The common primary and secondary chromosomal abnormalities in MM and their affected genes or chromosomes, frequency, risk stratification, and clinical prognosis are summarized in **Table 1**.

**Table 1.** Common primary and secondary chromosomal abnormalities and their prognostic outcomes in MM

**Chromosomal abnormality**	**Genes/chromosomes affected**	**Frequency (%)**	**Risk stratification**	**Prognosis**	**Remarks**	**References**
**Primary oncogenic events**						
Hyperdiploidy	Trisomies of odd-numbered chromosomes	50–60	Standard risk	Favorable Positive impact on OS and PFS	Trisomy 21-negative impact on OS Trisomy 3 or 5-positive impact on OS	[12,13,14,15,16,17]
IgH translocations		60			Occur as early as the MGUS stage Caused by errors in CSR (90%) and SHM (10%)	[12, 18, 19]
t(4;14)(p16;q32)	*FGFR3/ MMSET*	15	High risk	Unfavorable/neutral Negative impact on OS and PFS	Commonly developed from errors in CSR Increases rate of relapse Co-occurrence with trisomy 3 or 5 might improve prognosis	[10, 17, 20,21,22,23,24,25]
t(14;16)(q32;q23)	*c-MAF*	5–7	High risk	Unfavorable		[14, 19, 20, 26]
t(14;20)(q32;q11)	*MAFB*	1–2	High risk	Unfavorable		[14, 20, 27]
t(11;14)(q13;q32)	*CCND1*	15–20	Standard risk	Favorable/neutral	Commonly caused by SHM 40% RRMM response to Venetoclax High *MCL1*causes resistance to Venetoclax	[10, 28,29,30]
Others:						
t(12;14)(p13;q32)	*CCND2*	<1	Standard risk	Favorable/neutral		[19]
t(6;14)(p21;q32)	*CCND3*	2	Standard risk	Favorable/neutral		
t(8;14)(q24.3;q32)	*MAFA*	<1	High risk	Unfavorable		

**Secondary oncogenic events**						

del(1p)	*CDKN2C/ FAM46C/ FaF1*	30	High risk	Unfavorable Negative impact on OS and PFS	1p21 and 1p32 are the most frequently deleted regions	[31,32,33]
Gain (1q21)	*CKS1B/ PSMD4*	40	High risk	Unfavorable Negative impact on OS and PFS	Rarely found in MGUS Involves in disease progression Induces bortezomib resistance Worsens prognosis in cases with copy numbers ≥4	[34,35,36,37]
Monosomy 13/ del(13q)	*DIS3/ RB1*	50		Neutral	85% are monosomy; 15% are partial deletion Responds well to first-line therapy	[8, 32, 38,39,40]
del(17p)	*TP53*	5–10 NDMM 40 advanced MM	High risk	Unfavorable Negative impact on OS and PFS	Late event in pathogenesis Might be resistant to standard therapy	[11, 41, 42]
MYC translocation	*MYC/ IgK/ IgL/ IgH/ FOXO3/ FAM46C/ TXNDC5/ BMP6*	15 early-stage MM 50 advanced MM		Unfavorable/neutral Negative impact on OS and PFS	*IgH/MYC* or *IgK/MYC* are commonly associated with disease progression *IgL/MYC* adverse prognosis in NDMM Increases risk of progression from SMM to MM	[11, 12, 42,43,44,45,46,47,48,49]
Others						
del(11q)	*BIRC2/3*	7				[12, 32, 50, 51]
del(16q)	*WWOX/ CYLD*	35		Unfavorable		
del(14q)	*TRAF3*	38		Unfavorable		
del(12p)	*CDKN1B*			Unfavorable		
del(8p)	*TRAIL*			Unfavorable		

CSR, class-switch recombination; MGUS, monoclonal gammopathy of undetermined significance; MM, multiple myeloma; NDMM, newly diagnosed multiple myeloma; OS, overall survival; PFS, progression-free survival; RRMM, relapsed or refractory multiple myeloma; SHM, somatic hypermutation; SMM, smoldering multiple myeloma.

### Double and triple hits in MM

The co-occurrence of two or three high-risk genetic alterations is defined as double-/triple-hit MM. Based on the criteria stated in the IMWG, clinical outcomes of double-hit MM are similar regardless of whether they are classified as high-risk or low/standard-risk MM. Thus, defining the molecular features of double-hit MM is important to improve risk stratification and outcome prediction in MM. Double-hit MM is found in 1 of 33 newly diagnosed multiple myeloma (NDMM) [40]. According to Baysal et al. [52], the OS of MM patients with double-hit, single high-risk, and no high-risk MM were 6 months, 32 months, and 57 months, respectively. The same study found that the hazard ratio was worsened in triple-hit (7.30) than double-hit (5.55) and single high-risk (1.42) MM patients [52]. Another study revealed a close relationship between the co-occurrence of translocation t(4;14) and *TP53* mutation or del(17p) and *TP53* mutation, where they were found to confer poor prognosis and OS in the patients [53]. By using genome-wide NGS analysis, two genomic variables in double-hit MM were defined according to the background of International Staging System (ISS) III [54]. They were the biallelic inactivation of *TP53* and amplification (≥4 copies) of *CKS1B* (1q21). Biallelic inactivation of *TP53* has an adverse prognosis compared to wild-type or monoallelic inactivation of *TP53* [54]. Amplification of *CKS1B* has a shorter progression-free survival (PFS) and OS than a gain of 1q. There was no significant change in PFS when amplification of 1q co-occurred with biallelic *TP53*, del(17p), t(4;14), or t(14;16) [54]. Double/triple hits exhibited extra-high-risk features in MM, and the higher is the number of “hits,” the lower is the OS of the patients. In addition to the number of “hits,” the type of double/triple hits also significantly influences the prognosis outcomes and OS of the patients. Thus, patients presented with double/triple hits may require more intensive treatment than those with standard-risk MM, suggesting the importance of defining subtle genetic differences in patients using high-end technology to improve prognostic and treatment outcomes.

### GEP in MM

Transcriptome changes play a critical role in myelomagenesis as early as in the monoclonal gammopathy of undetermined significance (MGUS) stage [55]. In the past decades, GEP or microarray has emerged as one of the most popular approaches in the identification of transcriptome changes, molecular mechanisms, and pathways underlying human cancers. GEP has contributed to the characterization of disease subtypes, risk stratification, prognosis, and outcomes in MM patients. One of the most prominent results from GEP was the discovery of a 70-gene signature model by the University of Arkansas for Medical Sciences (UAMS) in 2007 [56]. The 70-gene signature was found to be superior in risk and prognostic stratification of NDMM. They found that most of the crucial genes related to disease progression were localized in chromosome 1. From the same study, 17-gene subsets for high-risk MM were also revealed. Among the important genes found in the 17-gene subset prediction model are *CKS1B*, *ASPM*, and *CTBS*.

Another significant study was reported by Broyl et al. [57]. They discovered seven unique clusters, which were associated with NDMM based on the gene expression profiles: i. translocation [MMSET/t(4;14), MAF/t(14;16)/t(14;20), cyclin D1/t(11;14), and cyclin D2/t(6;14)]; ii. hyperdiploidy (HY); iii. proliferation-associated genes (PR); iv. low percentage of bone disease (LB); v. overexpression of cancer testis antigens (CTAs); vi. NFκB pathway; and vii. overexpression of protein tyrosine phosphatases (PRLs) [57]. Among the seven clusters, three were newly identified (v–vii), while the rest were consistent with the UAMS classification [58].

The EMC-92 gene signature prediction model for high-risk MM was developed by another group of scientists from the Netherlands based on the findings from the GEP [59]. Survivin/*BIRC5*, *FGFR3*, and *STAT1* are the key genes in the EMC-92 model. Recent combination analysis of EMC-92, UAMS70, ISS, and FISH [t(4;14) & del(17p13)] has shown that EMC-92 gene classifier + ISS was the most superior marker for risk stratification in MM patients [59, 60]. This sheds light on the application of GEP in prognosis and risk prediction, replacing FISH, which is relatively expensive and laborious. FISH, however, can be used as a confirmation test if required.

## NGS

### NGS of MM

Although GEP has been successfully used to characterize MM into different clinicopathological subtypes and molecular signatures for diagnosis, staging, risk stratification, and prognostication of MM, data generated by GEP are inadequate to fully delineate the molecular biology of this highly genetically complex disease. In recent years, NGS has become a promising tool in the study of IgH translocations, V(D)J clonal rearrangements, IgH isotype, CNAs, and somatic mutations simultaneously in a more refined manner.

**Figure 2.**
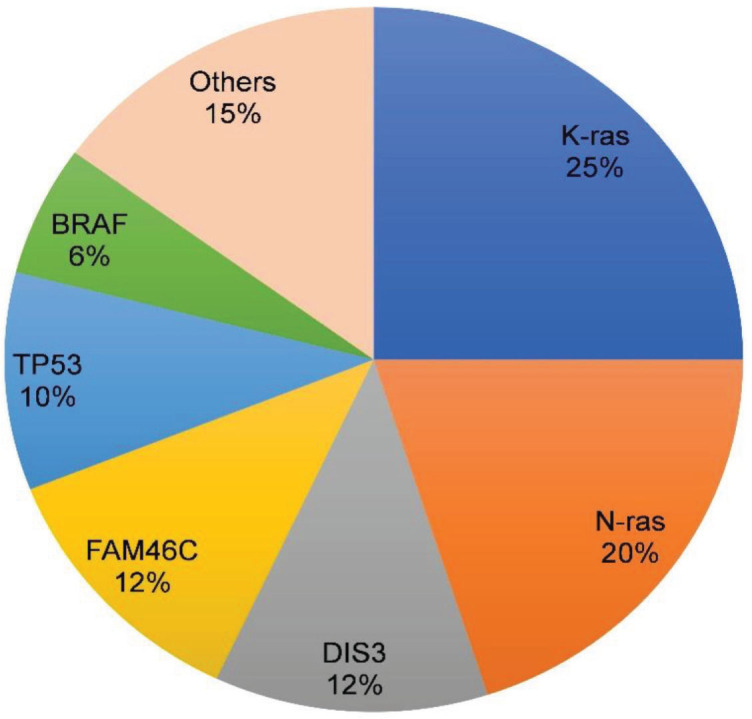
Recurrent gene aberrations and their frequency in MM. MM, Multiple myeloma.

The very first mutational profiles generated by NGS data in the past 10 years revealed that there is no specific or unique mutation in MM. The observation remains valid in the latest findings [61, 62]. To date, approximately 250 mutated genes have been found in MM, and about a quarter of them are identified as driver genes [62]. *N-Ras*, *K-Ras*, *DIS3*, *FAM46C*, *TP53,* and *BRAF* are considered the top recurrent or driver genes in MM, with a mutation frequency range between 6%–25% each (**Figure 2**) [53, 61, 63, 64]. Mutation in other driver genes such as *TRAF3*, *SP140*, *IRF4*, *JAK3,* and *PPGFRA* are rare with low recurrence rates (<5%) [65, 66]. Other newly identified potential driver genes are *IDH1*, *IDH2*, *HUWE1, KLHL6,* and *PTPN11* [67]. *N-Ras*, *K-Ras*, and *BRAF* are key components of the MAPK pathway [61, 68, 69]. Studies showed that the MEK-ERK signaling pathway was affected by *BRAF* and *RAS* mutations in >50% of the cases [70]. The most common site of N-Ras mutation is in codon Q61, while in K-Ras, it is in codons G12, G13, and Q61 [67]. On the other hand, *BRAF* mutation is predominantly seen in codon D594 (92%) in the t(14;16) group and *BRAF* V600E in the t(4;14) group [67]. The discovery of mutations in *BRAF* allowed many MM patients to benefit from *BRAF* inhibitor (vemurafenib) treatment [10].

**Figure 3.**
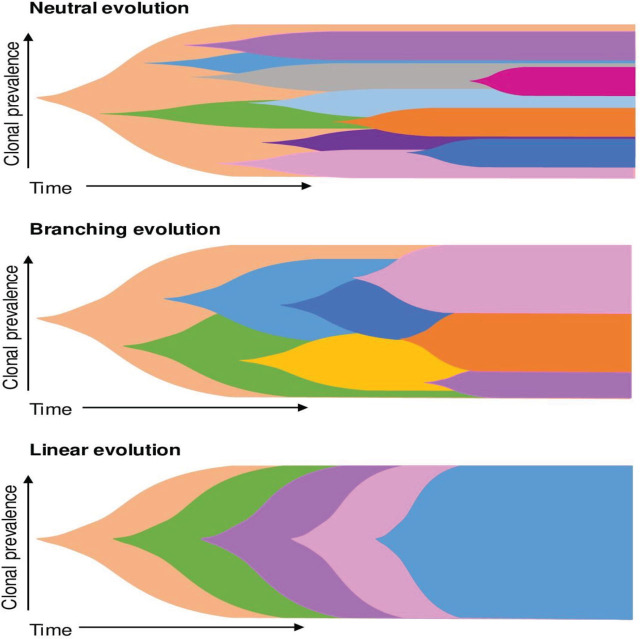
Main clonal evolution models in multiple myeloma. Each color represents a single subclone.

NGS findings not only confirm the heterogeneous complexity of MM but also show that most of the driver mutations are present in the subclonal population, and multiple mutations are detected in different genes within the same pathway, suggesting a diverse pattern of clonal evolution in MM, which evolves through space and time [10, 70]. The main clonal evolution in MM includes neutral evolution, branching evolution, and linear evolution [10, 71]. Neutral evolution occurs when all descendant subclones show a similar ability to survive under certain circumstances, and the evolutionary changes are not caused by selective pressure. Branching evolution is the early divergence of subclones with different mutations, which evolves further over time, possibly driven by the selective pressure of the bone marrow microenvironment and treatment, or inherent tumor characteristics, or both. In linear evolution, a single subclonal population is fully substituted by another highly adapted subclone [10, 71]. The main clonal evolution models in MM are visualized in **Figure 3**. Interestingly, recent NGS studies have shown that the emergence of multiple selective clonal sweeps derived from single cells can drive relapse even after 10 years of remission, suggesting the dormancy of resistant clones [72].

**Figure 4.**
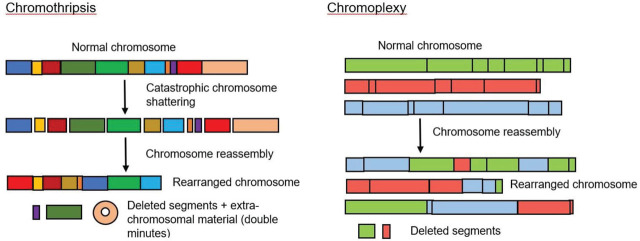
Complex chromosomal rearrangements in multiple myeloma: chromothripsis and chromoplexy.

Despite the discovery of driver gene mutations and clonal evolution in MM, another major contribution by NGS was the uncovering of complex SVs in MM. Complex SVs are usually difficult to detect using conventional sequencing approaches. Common complex SVs in MM include chromothripsis, chromoplexy, and multiple templated insertions [73]. Generally, MM patients present with at least one complex SV (80%) [38]. Chromothripsis, a form of chromoanagenesis, was first detected in chronic lymphoblastic leukemia (CLL) [74]. Chromothripsis is characterized by ten to hundreds of chromosomes breaking and rejoining in confined genomic regions in one or a few chromosomes (**Figure 4**). In contrast, chromoplexy involved a larger number of chromosomal rearrangements than chromothripsis (**Figure 4**). It was first identified in prostate cancer using whole-genome sequencing (WGS) [75]. Chromothripsis and templated insertions were found to be associated with clonal events, which occur early in the disease course, while chromoplexy is a late event that is possibly related to disease progression, drug resistance, and relapse [38]. There is a strong association between chromothripsis and adverse clinical outcomes in NDMM [76]. Templated insertions are less common than chromothripsis (19% vs 24%) [77]. It is characterized by focal gains bounded by translocations, resulting in the concatenation of amplified segments from two or more chromosomes into a continuous stretch of DNA, which is inserted back into any of the related chromosomes. SV hot spots frequently implicate known MM oncogenes or tumor suppressor genes such as *Ig*, *MYC, CCND1, MMSET, IRF4, MAP3K14, FAM46C, CDKN2C, CYLD*, and *SP140* [38, 78]. Recently, novel SVs involving critical genes in immune-based therapies *TNFRSF17, SLAMF7*, and *BCMA* were also revealed [78]. Patients who harbored SVs involving these genes tend not to respond to elotuzumab, BiTEs, or CAR-T cell therapy, or to their combination. NGS of MM also discovered various SVs hot spots in novel potential driver genes such as *IRF2*, *BTG2*, *SOX30*, *NEDD9*, *KLF13*, and *TNFRSF13B* [78].

NGS also contributes to the revelation of the activity-induced deaminase (*AID*)/apolipoprotein B mRNA editing enzyme and catalytic polypeptide-like (*APOBEC*) mutational signatures in MM. *AID* or *APOBEC* plays an important role in mediating somatic mutations and genomic instability in MM. The role of *AID* in initiating oncogenesis of MM has long been studied. *AID* is an essential regulator of somatic hypermutation and class-switch recombination of Ig genes. *AID* initiates C to U mismatches in single-stranded DNA and preferentially deaminates WRC/GYW hot spots (W=A/T, R=A/G, Y=C/T) [79]. *AID* is also a crucial mutator for *IgH*/*MYC* chromosomal translocation and oncogene mutation in MM [80]. So far, many *AID*-target driver genes have been identified. Among the most significant were *EGR1*, *IRF4*, *LTB*, *PIM1*, *TET2*, and *XBP1* [81]. Mutations in *AID*-target driver genes provide a selective growth advantage in the early phase of the disease [53].

On the other hand, *APOBEC* family proteins consist of 10 enzymes, namely, A1, A2, A3A, A3B, A3C, A3D, A3F, A3G, A3H, and A4. *APOBEC* is critical in mRNA editing and antiviral defense in the cells [82]. Dysregulation of *APOBEC* causes C>T, C>G, and C>A mutations in a TpC context and thereby resulting in copy number and mutational changes. In contrast to *AID*, *APOBEC* mutational activity is likely correlated with late events in disease progression [53]. High expression of *APOBEC* promotes DNA damage and increases mutational burden in MM (e.g., *APOBEC3G*) [82, 83]. Studies have shown that aberrant *APOBEC* expression is associated with translocations t(14;16)/*MAF* and t(14;20)/*MAFB* and a poor prognosis impact in MM [83]. However, only a small fraction of patients who presented with *MAF*/*MAFB*/*MAFA* translocations were related to high *APOBEC* activity (~23%), suggesting that other factors may be involved in regulating *MAF*-related translocations [84]. Co-occurrence of *APOBEC* activity and ISS stage III reduced OS in MM patients when compared to patients without the risk factors (53.8% vs 93.3%) [84]. Interestingly, *APOBEC* also triggers kataegis, a pattern of localized hypermutations characterized by C>T transitions at TpC dinucleotides. Kataegis has been observed at the *MYC*/*IgK* or *IgL* translocation breakpoints, implicating the co-occurrence of translocations and *APOBEC-*associated mutations in MM [85]. Suppression of *APOBEC* in MM cell lines was shown to reduce genomic and mutational changes by 30–65% [82]. A recent study that investigated the genomic spectrum of smoldering multiple myeloma (SMM) has shown that SMM patients with *APOBEC*-associated mutations demonstrated a shorter time of progression to MM [42]. In other words, the presence of *APOBEC* mutational signatures could be an indicator for higher risk of progression from SMM to symptomatic MM. Therefore, *APOBEC* inhibitors are potential therapeutic agents that can be used to suppress genome evolution in SMM or MM as a measure to prevent or delay the progression of the disease. However, this approach is still being developed. Nonetheless, due to the negative impact of *APOBEC* activity in prognostic and OS of MM patients, it is worth to include *APOBEC* analysis early on at the diagnosis stage to improve the treatment choice and efficacy in patients with high *APOBEC* activity.

### NGS of MGUS or SMM

The genomic spectrum of MGUS and SMM has been studied using NGS as well, although they are not as much as in MM. MGUS is a benign noncancerous condition, with a ~1% progression rate to MM per year. In contrast, SMM is a precancerous or early-stage MM, with a 10% annual progression risk to active MM in the first 5 years of diagnosis [86].

By comparing NGS data from paired SMM and MM from the same individual, Bolli et al. [53] postulated that most of the driver mutations such as hyperdiploidy, *IgH* translocations, 1q gain, and del(13q) in SMM were clonal and retained in MM. They found 2 unique clusters during the progression of SMM into MM: the static progression model, in which the same subclonal architecture in SMM was retained in MM; spontaneous evolution cluster, where the additional mutation in subclonal composition was acquired during disease progression [53]. Their findings support the clonal evolution patterns that have been described before [10]. Importantly, their results suggest different management and treatment strategies for the two clusters. Another study was published in 2020, which described the genomic signatures of SMM and its association with risk progression to MM [42]. The group analyzed the genomic profiles of 214 SMM patients using whole-exome sequencing (WES) or deep-targeted sequencing approaches. Their study findings were consistent with Bolli et al. [53], which demonstrated that most of the driver mutations previously defined in MM were already present in SMM patients. These driver mutations were also identified as independent risk factors of progression to MM [42]. In addition, their findings have shown that SMM, which presented with genetic alteration related to the MAPK pathway, DNA repair pathway, or *MYC*, has a higher risk of progression to MM.

In addition to comparing genomic data between SMM and MM, WGS has also been used to study the differences in the genomic landscape and temporal acquisition of myeloma-associated genomic events between clinically stable and progressive myeloma precursor conditions (MGUS and SMM) [87]. Compared with the clinically progressive myeloma precursor condition, the clinically stable myeloma precursor condition was associated with late initiation of the first clonal copy number alteration in patients’ life and absence or lower number of mutations in driver genes (genes involved in MAPK and NF-κB pathways, *TP53*), aneuploidy (gain of 1q, deletion of 6q, gain of 8q24, deletion of 8p, and deletion of 16q), templated insertions, chromothripsis, and *APOBEC-*associated mutational activity.

## Conclusions

In summary, advances in technology have expanded our knowledge of tumor heterogeneity, mutational landscape, clonal composition, and dynamic evolution of MM. Evidence has confirmed that myeloma is a highly heterogeneous disease, and one single treatment regimen does not fit all MM patients. Thus, precision medicine will become inevitably important in future myeloma therapy. NGS, which allows the analysis of the full spectrum of recurrent mutations and chromosomal abnormalities in MM, can become a powerful tool toward precision medicine in the treatment of MM. Importantly, NGS also enables rapid and accurate detection of clonal and subclonal mutations, which present at low variant allele frequencies in patients. Many of these subtle genetic changes have a significant influence on the drug efficacy and prognosis outcomes of the patients. NGS has been proven to provide significant clinical benefits and should be transitioned from research to clinical use. However, NGS is laborious, time-consuming, and remains expensive even today, thus preventing its routine use in clinical settings. Malaysia, one of the developing countries in Southeast Asia, also faces the same challenges. So far, we are relying on conventional karyotyping and FISH for routine diagnosis, risk assessment, and therapeutic selection of MM patients. As discussed earlier, karyotyping and FISH have limited resolution and therefore are inadequate to capture the complexity of the MM genome. As technological advances will continue to push NGS toward lower costs with more user-friendly methodologies and data analysis pipelines, we hope that NGS will be integrated into routine clinical practice at diagnosis/relapse to guide disease management and allow for precision medicine in future.
